# Lactic acidosis secondary to metformin overdose: a case report

**DOI:** 10.1186/1752-1947-6-230

**Published:** 2012-08-02

**Authors:** Simon Timbrell, Gary Wilbourn, James Harper, Alan Liddle

**Affiliations:** 1 Intensive Care Unit, Lincoln County Hospital, Lincoln, LN2 5QY, UK; 2 Queens Medical Centre, Nottingham University Medical School, Nottingham, UK

## Abstract

**Introduction:**

Metformin is a commonly used treatment modality in type 2 diabetes mellitus, with a well documented side effect of lactic acidosis. In the intensive care setting lactate and pH levels are regularly used as a useful predictor of poor prognosis. In this article we highlight how high lactate levels are not an accurate predictor of mortality in deliberate metformin overdose.

**Case presentation:**

We present the case of a 70-year-old Caucasian man who took a deliberate metformin overdose of unknown quantity. He had a profound lactic acidosis at presentation with a pH of 6.93 and a lactate level of more than 20mmol/L. These figures would normally correspond with a mortality of more than 80%; however, with appropriate management this patient’s condition improved.

**Conclusion:**

We provide evidence that the decision to treat severe lactic acidosis in deliberate metformin overdose should not be based on arterial lactate and pH levels, as would be the case in other overdoses. We also demonstrate that appropriate treatment with hemodiafiltration and 8.4% sodium bicarbonate, even in patients with a very high lactate and low pH, can be successful.

## Introduction

Metformin is a biguanide typically used as a first line drug for the treatment of type 2 diabetes mellitus. Its chief modes of action are reduced absorption of glucose from the gastrointestinal tract, decreased hepatic gluconeogenesis and increased peripheral utilization of glucose [1]. All of these actions lower plasma levels of glucose.

Commonly reported side effects are predominantly gastrointestinal and include: nausea, vomiting and abdominal pain. Lactic acidosis is a well-recognized consequence of toxicity and is often associated with concurrent renal or hepatic insufficiency and in an overdose. The mechanism of hyperlactatemia in metformin toxicity is not fully understood.

The normal range for arterial lactate is 0.5 to 1.6mmol/L. Elevated levels are a common finding within critical care patients. Levels exceeding 2 to 3mmol/L have been shown to be an independent marker for mortality in critical care patients with sepsis and in amitryptyline overdose [2]. Much higher mortality rates of nearly 70% have been independently associated with lactate levels of >3.5 [3]. This compelling evidence of excessive mortality should, however, not be extrapolated to all causes.

We present a case of deliberate metformin overdose in which a patient presenting with an arterial lactate of more than 20mmol/L.

## Case presentation

A 70-year-old Caucasian man with type 2 diabetes mellitus presented to the emergency department three hours after a suicide attempt in which an unknown quantity of metformin was ingested.

At presentation he was vomiting, severely agitated, confused and complaining of abdominal pain. He had a heart rate of 100 beats per minute, blood pressure of 150/80mmHg and a respiratory rate of 20. His cardio-respiratory system was normal; his abdomen was soft and non-tender.

Arterial blood gas analysis on admission showed a profound lactic acidosis: His temperature was 37.0°C. The results of his blood analysis are as follows: pH 6.93 (7.38 to 7.42); pCO_2_ 2.1kPa (4.8 to 5.8); pO_2_ 30.4kPa mmol/L (12 to 14); actual bicarbonate 3mmol/L (22 to32); base excess −27.7mmol/L (−2 to 2); and lactate 20.0mmol/L (0.5 to 2.2). This value is the upper limit of our machine.

Alongside the profound acidaemia, he was anuric with acute kidney injury and a creatinine of 215μmol/L. Table 1 shows his blood results at presentation.

**Table 1:** Blood results at presentation

**Clinical Chemistry Parameters**	**Patient’s Values**	**Normal Ranges**
Sodium	147	133 to146mmol/L
Potassium	4.1	3.5 to 5.3mmol/L
Urea	8.9	2.5 to 7.8mmol/L
Creatinine	208	64 to 110μmol/L
Bilirubin	11	0 to 21μmol/L
AST	29	5 to 34μ/L
ALT	33	0 to 37μ/L
Alkaline phosphatase	55	3 to 130μ/L
Total protein	80	60 to 80 g/L
C-Reactive Protein	4.5	0 to 8 mg/L
Hemoglobin	15.1	13.5 to 16.9g/dL
Platelets	290	140 to 400 × 10^9^/L
White Cell Count	14.40	4.5 to 13.0 × 10^9^/L
Neutrophils	11.40	2 to 7.5 × 10^9^/L

ALT, alanine aminotransferase; AST, aspartate aminotransferase.

He was admitted to the intensive care unit and started on continuous veno-venous hemodiafiltration (CVVHDF) with an average blood flow rate of 150mls/hour (varying between 100 to 180mls/hour) for the 48 hours described in Table 2 and an hourly replacement flow rate of 1500mls/hour (varying between 1000 to 2000mls/hour) and dialysate replacement with 8.4% sodium bicarbonate with an average rate of 1500mls/hour (varying between 1000 to 2000mls/hour) .

**Table 2:** Response to hemofiltration

**Date**	**0 hours**	**6 hours**	**24 hours**	**48 hours**
pH (7.38 to 7.42)	6.93	7.02	7.36	7.37
pCO_2_ (4.8 to 5.8kPa)	2.1	4.2	5.3	4.8
pO2 (12 to 14kPa)	30.4	15.6	13.3	12.4
Bicarbonate (22 to 32mmol/l)	3	8	23	21
Base Excess (−2 to 2mmol/l)	−27.7	−21.7	−2.7	−3.9
Inspired oxygen (%)	60	29	60	40
Creatinine (64 to 110μmol/L)	215	151	167	138
Lactate (0.5 to 2.2mmol/L)	>20	>20	13.1	4.0

On initiating hemofiltration he developed uncontrolled atrial fibrillation with cardiovascular compromise requiring amiodarone infusion and vasopressor support.

Despite these initial complications, Table 2 highlights how quickly the lactic acidosis responded to hemofiltration.

Despite the clearance of the lactate with CVVHDF his renal function did not improve. He was offered continued renal support under the care of the renal physicians which he refused. He subsequently developed a hospital-acquired pneumonia, for which he refused active treatment, and died.

## Discussion

Lactic acid is produced when there is inadequate tissue oxygenation for aerobic respiration and lactate is produced as a by-product of anaerobic respiration catalyzed by the actions of lactate dehydrogenase on pyruvate, as shown below:

(1) $$Pyruviate+NAD(nicotinamide\;adenine\;dinocleotide)H+H^{+}\Leftrightarrow Lactate+NAD^{+}$$

Hyperlactemia is defined as an abnormally high lactate level between 2 to 5mmol/L whereas lactic acidosis is defined as a lactate level of more than 5mmol/L and a pH of less than 7.34.

There are two isomeric forms of lactate: D-lactate, a byproduct of bacterial metabolism, and L-lactate, produced in human tissues by anaerobic respiration.

Cohen and Woods [4] divided the causes of lactic acidosis into 2 categories:

Type A lactic acidosis in which there is clinical evidence of inadequate tissue perfusion or oxygenation.

This includes:

Anaerobic muscular activity (for example, exercising)

Tissue hypoperfusion (for example, septic shock)

Reduced tissue oxygen delivery or utilization (for example, severe anaemia), and

Type B lactic acidosis in which there is no clinical evidence of poor tissue perfusion and this is further subdivided into:

B1 - Associated with underlying diseases (for example, ketoacidosis)

*B2* – Associated with several classes of drugs and toxins (for example, biguanides, salicylates, isoniazid and alcohol)

*B3* - Associated with inborn errors of metabolism (for example, pyruvate dehydrogenase deficiency).

Metformin-associated lactic acidosis is relatively rare occurring in 0.03 cases per 1000 patient-years [5]. It refers to any case of lactic acidosis in a patient taking metformin where no other lactate-inducing mechanisms, such as sepsis, are involved. In the majority of cases, the contribution of metformin is secondary to patient factors, such as renal insufficiency, liver disease or low cardiac output states. Although secondary, metformin is significant. The presence of metformin-associated hyperlactatemia in critical care patients has been associated with a mortality greater than 30% [6].

The patient in this scenario was suffering from metformin-induced lactic acidosis. This specifically refers to a situation in which the high lactate level cannot be explained by any major risk factor other than excessive metformin intake and is, therefore, a type B2 lactic acidosis. The difference being that metformin accumulation may coexist with other factors in metformin-associated lactic acidosis contributing to the pathogenesis of lactic acidosis, whereas in metformin-induced lactic acidosis, the lactic acidosis is purely caused by excessive amounts of metformin.

Deliberate overdose with oral hypoglycemic agents is a rarer form still of metformin-induced lactic acidosis. A literature review using the PubMed database only highlighted a few case reports and small case series, demonstrating how rare an occurrence lactic acidosis due to metformin overdose is. Further evidence of the unusual nature of the case comes from a five-year review of toxic exposures reported to US poison control centers. Within the review only 4072 out of nearly 11 million exposures involved metformin, corresponding to less than one in 2500 toxic overdoses [7].

Previous case reports of deliberate metformin overdose show how individuals with an excessively high lactate level, that would normally be considered incompatible with life, managed to survive. Gjedde *et al*.’s [8] case report demonstrates this, as an individual deliberately ingested 63 grams of metformin and survived despite having an initial lactate of 17.7mmol/L. A case series by Teale *et al*. [9] described how individuals with severe metabolic acidosis, the worse being a pH of 6.9 and lactate level of 29mmol/L, survived with appropriate management.

The previously published data agreed that the appropriate treatment for metformin overdose involves bicarbonate hemodialysis [8-11].

Correcting the metabolic acidemia is of paramount importance in metformin-induced lactic acidosis. Sodium bicarbonate is often the obvious method; however, it is not always effective due to lactate being produced at such a rate that even at high doses, sodium bicarbonate fails to sustain a normal blood pH. Dichloroacetate and tri-hydroxymethyl aminomethane are other options that have been described [11].

Hemodialysis via veno-venous hemodiafiltration with bicarbonate replacement fluid is deemed more effective, as the metformin is also cleared via the filter. This however requires the patient to be hemodynamically stable. Another way to encourage metformin excretion is to use furosemide to induce a diuresis which leads to increased excretion of unchanged metformin in the urine.

In this case the patient received CVVHDF with 8.4% bicarbonate sodium, which quickly resolved his lactic acidosis as shown in Table 2.

The association between lactic acidosis and a poor prognosis has been known for decades. Bakker and Jansen even suggested that lactate was a better indicator of patient outcome than measuring observations and many intensivists use it as a guide to patient mortality [12]. Manini *et al*. demonstrated how serum lactate levels were a good prognostic marker when used to predict drug overdose mortality [13].

Given the patient’s initial high lactate level traditional thoughts and evidence would have predicted a very high mortality. Indeed, using the Simplified Acute Physiology Score our patient had a predicted mortality of nearly 80%.

This case highlights how the measurement of arterial pH and lactate is not such an accurate clinical indicator in metformin-induced lactic acidosis. This is supported by Vecchio and Protti’s recent research which suggests that arterial pH and lactate are not good indicators of mortality in patients with metformin-associated lactic acidosis [14]. In their case series involving metformin overdose the average arterial pH at admission was 6.75 ± 0.13 and lactate was 19 ± 5mmol/L. These patients had a Simplified Acute Physiology Score II of 88 ± 23 and their predicted mortality was 96%; however, survival rates were much higher at 50%. This work also compared similarly severe lactic acidosis from non-metformin associated causes and of the 31 patients, none survived. This is further supported by the work of Nyirenda *et al*. who demonstrated an actual mortality of 10% in metformin overdose against a predicted mortality of 55% [15].

A proposed mechanism for why metformin-induced lactic acidosis with such a high lactate level and low pH may be more reversible is highlighted in further work by Protti *et al*. where they suggest biguanides induce lactic acidosis by impairing hepatocyte mitochondrial function [16]. Lactate production is, therefore, an epiphenomenon due to inhibition of mitochondrial function and this may explain why rapid removal of the offending metformin via CVVHDF can effectively treat extremely high lactate levels [16].

## Conclusion

This case report highlights that although there is much evidence showing that hyperlactatemia is a very poor prognostic indicator, this does not necessarily apply to patients who take a deliberate metformin overdose.

Based on this case and recent research, the decision to treat (or not to treat) a patient with severe lactic acidosis with metformin ingestion cannot be based on the arterial lactate and pH, as would be the case in sepsis or amitriptyline overdose. This scenario highlights that appropriate management with continuous veno-venous hemodiafiltration and 8.4% sodium bicarbonate can be used to treat patients successfully who have a perceived high mortality.

We would, therefore, recommend that all patients with metformin-induced lactic acidosis must be treated no matter how high the serum lactate and low the pH, as renal replacement therapy can efficiently remove metformin and sodium bicarbonate can correct the acidosis.

## Consent

Written informed consent for publication from the patient’s next of kin could not be obtained despite all reasonable attempts. The patient had no children or living family and declined to identify any friends that should be informed of his admission who could act as a representative. The case is important to public health and every effort has been made to protect the identity of our patient. There is no reason to believe that our patient’s next of kin would object to publication.

## Competing interests

The authors declare that they have no competing interests.

## Authors’ contributions

ST collected and interpreted the patient data. ST and JH were involved in the literature review and writing of the article. GW was involved in writing and editing the article. AL edited the article. All authors read and approved the final manuscript.

## Authors’ information

ST, Foundation Year 1, Intensive Care Unit, Lincoln County Hospital. GW, Specialist Registrar (ST7), Intensive Care Unit, Lincoln County Hospital. JH, Final Year Medical, Student University of Nottingham. AL, Intensive Care Consultant, Intensive Care Unit, Lincoln County Hospital.
